# Supplementary material for: A novel deep learning method for large-scale analysis of bone marrow adiposity using UK Biobank Dixon MRI data
Source: Comput Struct Biotechnol J. 2023 Dec 27;24:89–104. doi: 10.1016/j.csbj.2023.12.029 (PMC10806280; doi:10.1016/j.csbj.2023.12.029)
Supplement: Supplementary file 1 — Supplementary material [file mmc1.pdf]

SUPPLEMENTAL DIGITAL CONTENT

A novel deep learning method for large-scale analysis of bone marrow adiposity using UK Biobank Dixon MRI data

Morris and Wang *et al*.

SUPPLEMENTAL DIGITAL CONTENT

The Supplemental Digital Content includes one data file (.xlsx) and five tables, as follows:

Supplemental Data – An .xlsx spreadsheet describing PheCodes for skeletal conditions, which were assessed among the validation cohort because these conditions might affect skeletal morphology and thereby influence deep learning segmentation. For each PheCode the following are shown: corresponding ICD10 and ICD9 codes; the disease description from each coding system; the prevalence among participants in the validation cohort; and the prevalence among participants giving faulty or normal segmentation outputs for each skeletal site. This demonstrates that PheCode prevalence is similar among faulty and normal segmentations, confirming that skeletal pathologies are not a systematic cause of faulty segmentation outputs from this cohort.

Supplemental Table 1 – Univariable associations between spine BMD and BMFF, Age, BMI or adiposity traits.

Supplemental Table 2 – Univariable associations between femoral head BMD and BMFF, Age, BMI or adiposity traits.

Supplemental Table 3 – Univariable associations between total hip BMD and BMFF, Age, BMI or adiposity traits.

Supplemental Table 4 – Univariable associations between femoral shaft BMD and BMFF, Age, BMI or adiposity traits.

Supplemental Table 5 – Univariable associations between BMFF at each region and Age, BMI or adiposity traits.

Dependent	Explanatory	Sex	β (95% CIs)	Adj. R^2	*P* (Exp)	*P* (Exp*Sex)
BMD Spine	BMFF Spine	Both	**-0.004 (-0.006, -0.003)**	**0.390**	**1.09E-09**	0.431
	Age	Both	0 (-0.004, 0.003)	0.352	0.930	**0.021**
		F	-0.003 (-0.007, 0.001)	0.003	0.116	**-**
		M	0.005 (-0.001, 0.012)	0.006	0.108	**-**
	BMI	Both	**0.023 (0.017, 0.029)**	**0.395**	**1.61E-12**	**7.827E-05**
		F	**0.015 (0.008, 0.022)**	**0.036**	**3.28E-05**	**-**
		M	**0.043 (0.03, 0.055)**	**0.137**	**1.25E-10**	**-**
	VAT mass (kg)	Both	**0.081 (0.054, 0.109)**	**0.380**	**1.01E-08**	0.840
	Total fat %	Both	0.002 (0, 0.004)	0.354	0.119	0.438
	Android fat %	Both	**0.002 (0.001, 0.004)**	**0.369**	**9.25E-06**	0.904
	Gynoid fat %	Both	-0.002 (-0.004, 0)	0.354	0.096	0.208
	Trunk fat %	Both	**0.003 (0.001, 0.004)**	**0.365**	**8.84E-05**	0.716
	Legs fat %	Both	**-0.004 (-0.006, -0.001)**	**0.362**	**6.78E-04**	0.276

Supplemental Table 1 – Univariable associations between spine BMD and BMFF, Age, BMI or adiposity traits. To test if the explanatory-dependent relationship differs between males and F, a linear model was first analysed across both sexes, with sex included as an interacting variable. Beta coefficients are shown (with lower and upper 95% CIs in brackets), followed by the adjusted R^2 (Adj. R^2) and unadjusted *P* value for each explanatory variable (*P* Exp). *P* values were also calculated for the Explanatory*Sex interaction (*P* Exp*Sex); if significant, additional linear models were analysed in females (F) and males (M) separately. Because 13 correlations were assessed, the Bonferroni-adjusted alpha level for *P* (Exp) is 0.05/13 = 0.0038. Significant explanatory-dependent relationships are highlighted in bold.

Dependent	Explanatory	Sex	β (95% CIs)	Adj. R^2	*P* (Exp)	*P* (Exp*Sex)
BMD Femoral Neck	BMFF Femoral Head	Both	**-0.022 (-0.026, -0.018)**	**0.259**	**2.19E-21**	0.566
	BMFF Total Hip	Both	**-0.015 (-0.018, -0.012)**	**0.244**	**1.76E-20**	0.340
	BMFF Spine	Both	**-0.004 (-0.006, -0.003)**	**0.201**	**6.73E-12**	0.886
	Age	Both	-0.002 (-0.005, 0.001)	0.142	2.58E-01	0.809
	BMI	Both	**0.014 (0.008, 0.02)**	**0.167**	**2.17E-06**	0.070
	VAT mass (kg)	Both	0.021 (-0.005, 0.046)	0.143	1.13E-01	0.572
	Total fat %	Both	-0.001 (-0.002, 0.001)	0.141	4.38E-01	0.438
	Android fat %	Both	0 (-0.001, 0.001)	0.140	7.60E-01	0.381
	Gynoid fat %	Both	-0.002 (-0.004, 0)	0.144	8.71E-02	0.693
	Trunk fat %	Both	0 (-0.001, 0.001)	0.140	7.68E-01	0.553
	Legs fat %	Both	**-0.003 (-0.004, -0.001)**	**0.149**	**7.63E-03**	0.323

Supplemental Table 2 – Univariable associations between femoral head BMD and BMFF, Age, BMI or adiposity traits. Linear models were established as described for Supplemental Table 1. Because 11 correlations were assessed, the Bonferroni-adjusted alpha level for *P* (Exp) is 0.05/11 = 0.0045. Significant explanatory-dependent relationships are highlighted in bold.

Dependent	Explanatory	Sex	β (95% CIs)	Adj. R^2	*P* (Exp)	*P* (Exp*Sex)
BMD Total Hip	BMFF Femoral Head	Both	**-0.023 (-0.027, -0.018)**	**0.336**	**1.12E-19**	**0.838**
	BMFF Total Hip	Both	**-0.017 (-0.02, -0.014)**	**0.340**	**1.065E-23**	0.059
	Age	Both	-0.002 (-0.005, 0.001)	0.234	0.226	0.770
	BMI	Both	**0.022 (0.016, 0.028)**	**0.282**	**3.044E-12**	0.111
	VAT mass (kg)	Both	**0.044 (0.017, 0.071)**	**0.242**	**0.002**	0.979
	Total fat %	Both	0 (-0.002, 0.002)	0.232	0.929	0.175
	Android fat %	Both	0.001 (0, 0.002)	0.235	0.091	0.225
	Gynoid fat %	Both	-0.002 (-0.004, 0)	0.236	0.043	0.248
	Trunk fat %	Both	0.001 (0, 0.002)	0.235	0.106	0.240
	Legs fat %	Both	**-0.003 (-0.005, -0.001)**	**0.244**	**6.93E-04**	0.115

Supplemental Table 3 – Univariable associations between total hip BMD and BMFF, Age, BMI or adiposity traits. Linear models were established as described for Supplemental Table 1. Because 10 correlations were assessed, the Bonferroni-adjusted alpha level for *P* (Exp) is 0.05/10 = 0.005. Significant explanatory-dependent relationships are highlighted in bold.

Dependent	Explanatory	Sex	β (95% CIs)	Adj. R^2	*P* (Exp)	*P* (Exp*Sex)
BMD Femoral shaft	BMFF Femoral Diaphysis	Both	**-0.015 (-0.018, -0.012)**	**0.285**	**2.20E-24**	0.766
	Age	Both	-0.004 (-0.008, 0.001)	0.152	0.086	0.439
	BMI	Both	**0.024 (0.016, 0.032)**	**0.191**	**1.319E-09**	0.337
	VAT mass (kg)	Both	**0.046 (0.012, 0.08)**	**0.156**	**0.008**	0.983
	Total fat %	Both	0 (-0.003, 0.002)	0.149	0.870	0.419
	Android fat %	Both	0.001 (0, 0.002)	0.151	0.149	0.355
	Gynoid fat %	Both	-0.002 (-0.005, 0)	0.153	0.062	0.579
	Trunk fat %	Both	0.001 (0, 0.003)	0.151	0.153	0.409
	Legs fat %	Both	**-0.004 (-0.007, -0.002)**	**0.161**	**0.001**	0.427

Supplemental Table 4 – Univariable associations between femoral shaft BMD and BMFF, Age, BMI or adiposity traits. Linear models were established as described for Supplemental Table 1. Because 9 correlations were assessed, the Bonferroni-adjusted alpha level for *P* (Exp) is 0.05/9 = 0.0056. Significant explanatory-dependent relationships are highlighted in bold.

Dependent	Explanatory	Sex	β (95% CIs)	Adj. R^2	*P* (Exp)	*P* (Exp*Sex)
BMFF Spine	**Age**	Both	**0.269 (0.08, 0.459)**	**0.127**	**0.005**	0.542
	BMI	Both	0.219 (-0.138, 0.576)	0.119	0.229	0.425
	**VAT mass (kg)**	Both	**5.331 (3.833, 6.829)**	**0.177**	**6.640E-12**	0.549
	**Total fat %**	Both	**0.372 (0.267, 0.476)**	**0.176**	**6.492E-12**	0.075
	**Android fat %**	Both	**0.226 (0.167, 0.285)**	**0.185**	**1.14E-13**	0.157
	**Gynoid fat %**	Both	**0.241 (0.129, 0.352)**	**0.140**	**2.53E-05**	0.078
	**Trunk fat %**	Both	**0.287 (0.214, 0.36)**	**0.188**	**3.73E-14**	0.214
	Legs fat %	Both	0.141 (0.03, 0.252)	0.125	0.013	**0.011**
		F	0.061 (-0.06, 0.183)	0.000	0.323	**-**
		M	**0.4 (0.159, 0.641)**	**0.037**	**0.001**	**-**
BMFF Femoral Head	Age	Both	0.047 (-0.008, 0.102)	0.130	0.093	**0.043**
		F	0.088 (0.014, 0.278)	0.162	0.019	**-**
		M	-0.031 (-0.109, 0.047)	-0.002	0.435	**-**
	BMI	Both	-0.041 (-0.146, 0.064)	0.127	0.445	0.361
	VAT mass (kg)	Both	0.106 (-0.563, 0.351)	0.127	0.649	0.068
	Total fat %	Both	0.026 (-0.006, 0.059)	0.129	0.110	**0.014**
		F	0.051 (0.01, 0.092)	0.012	0.015	-
		M	-0.04 (-0.092, 0.012)	0.006	0.128	-
	Android fat %	Both	0.003 (-0.016, 0.021)	0.126	0.781	0.093
	Gynoid fat %	Both	0.041 (0.007, 0.074)	0.134	0.018	**0.010**
		**F**	**0.067 (0.024, 0.109)**	**0.021**	**0.002**	-
		M	-0.033 (-0.087, 0.022)	0.002	0.237	-
	Trunk fat %	Both	0.009 (-0.014, 0.031)	0.127	0.456	**0.034**
		F	0.023 (-0.005, 0.052)	0.004	0.110	-
		M	-0.032 (-0.067, 0.004)	0.009	0.084	-
	Legs fat %	Both	0.045 (0.013, 0.078)	0.136	0.007	0.142
BMFF Total Hip	Age	Both	0.075 (-0.001, 0.15)	0.071	0.048	0.297
	**BMI**	Both	**-0.235 (-0.374, -0.096)**	**0.081**	**9.63E-04**	0.864
	VAT mass (kg)	Both	-0.657 (-1.264, -0.05)	0.073	0.034	0.852
	Total fat %	Both	0.007 (-0.035, 0.049)	0.066	0.743	0.634
	Android fat %	Both	-0.008 (-0.031, 0.016)	0.066	0.527	0.940
	Gynoid fat %	Both	0.033 (-0.012, 0.077)	0.069	0.149	0.466
	Trunk fat %	Both	-0.005 (-0.035, 0.024)	0.066	0.726	0.772
	Legs fat %	Both	0.036 (-0.008, 0.08)	0.069	0.106	0.837
BMFF Femoral Diaphysis	Age	Both	0.107 (-0.001, 0.216)	0.048	0.053	0.625
	BMI	Both	-0.133 (-0.337, 0.072)	0.045	0.202	0.783
	**VAT mass (kg)**	Both	**-1.318 (-2.197, -0.438)**	**0.056**	**0.003**	0.096
	**Total fat %**	Both	-0.079 (-0.141, -0.018)	0.052	0.012	**0.009**
		F	**-0.135 (-0.211, -0.059)**	**0.027**	**5.10E-04**	**-**
		M	0.04 (-0.066, 0.146)	-0.002	0.456	**-**
	**Android fat %**	Both	**-0.067 (-0.101, -0.032)**	**0.063**	**1.597E-04**	**0.018**
		F	**-0.095 (-0.138, -0.053)**	**0.043**	**1.298E-05**	**-**
		M	-0.007 (-0.066, 0.052)	-0.004	0.823	**-**
	Gynoid fat %	Both	0.019 (-0.046, 0.085)	0.043	0.562	**0.034**
		F	-0.028 (-0.109, 0.053)	-0.001	0.496	**-**
		M	0.125 (0.013, 0.236)	0.015	0.029	**-**
	**Trunk fat %**	Both	**-0.081 (-0.124, -0.038)**	**0.062**	**2.183E-04**	**0.015**
		F	**-0.118 (-0.171, -0.065)**	**0.043**	**1.442E-05**	**-**
		M	-0.004 (-0.077, 0.07)	-0.004	0.920	**-**
	**Legs fat %**	Both	0.035 (-0.029, 0.1)	0.044	0.286	**0.008**
		F	-0.014 (-0.09, 0.062)	-0.002	0.725	**-**
		M	**0.191 (0.066, 0.316)**	**0.032**	**0.003**	**-**

Supplemental Table 5 – Univariable associations between BMFF at each region and Age, BMI or adiposity traits. Linear models were established as described for Supplemental Table 1. For each dependent variable, Bonferroni-adjusted alpha levels for *P* (Exp) are as follows: BMFF Spine, 0.005 (10 comparisons); BMFF femoral head, 0.0031 (16 comparisons); BMFF total hip, 0.00625 (8 comparisons); BMFF femoral diaphysis, 0.0028 (18 comparisons). Significant explanatory-dependent relationships are highlighted in bold.
